# Book Review: Facet Theory and the Mapping Sentence: Evolving Philosophy, Use and Application

**DOI:** 10.3389/fpsyg.2019.00468

**Published:** 2019-03-19

**Authors:** Paul M. Capobianco

**Affiliations:** Marketing Communications, Emerson College, Boston, MA, United States

**Keywords:** facet, theory, research, qualitative, theoretical, mapping, sentence, Hackett

In *Facet Theory and the Mapping Sentence* Paul M. W. Hackett makes a case for facet theory's ability to describe the complexity of the world and the categorial implications of such investigations. Even if we are familiar with facet theory, its integral instrument the mapping sentence is introduced to us anew in previously unexplored qualitative contexts. Ultimately, Hackett's non-orthodox applications of facet theory involving qualitative data do not seem heretical, in part, because facet theory is first presented as a plausible and enlightening way to see the world backed by centuries of philosophical precedent and recent developments including neuroscience literature. What is more these overviews are succinct and contribute in a clear way to an inspiring, verisimilar worldview. We are left willing to accept the idea that the rigor of mapping sentences could be useful not only as a research tool but also for areas as diverse as therapy and making fine art. In each case the purported usefulness and importance of using mapping sentences is stressed. The effect is intellectually satisfying and rhetorically effective because at each turn what we come to accept as the truth of facet theory is specially and coherently magnified by another display of its kaleidoscopic brilliance.

One of Hackett's more unique takes on what facet theory is and what it could mean to embrace it is distilled in what he calls the “facet theoretical imagination.” The mapping sentence is presented as an extension of this imagination: the embodiment of a stance that structures behavior and experience in terms of discrete categories or facets within which are subunits or elements meant to exhaustively account for each facet's variations. Hackett sees in this stance the opportunity to “envisage more complex events and to develop more multifarious awareness” ((Hackett, 2014), p. 62). Instead of superimposing a rigid, prescriptive lens over the research domain facet theory achieves a dynamic kind of clarity because the lens through which the user of the mapping sentence perceives the domain and conducts their research is carved by the context and respondents that compose the domain itself. Whilst static, the mapping sentence's inherent guidance for its own use and evolution draws out the real-life bustle of a domain over time where use improves performance.

A more realistic analogy is palate refinement. As this takes place flavors emergent from interactions with the food in the forms of taste and smell are repeatedly and purposefully categorized and subsequently more accurately identified in the context of its relationships with other flavors. Eventually attention to palate development will reveal deeper, longer, orchestral experiences. Moreover, flavors such as almond can be differentiated from cherry, vanilla, etc. The practice of palate development allows not only recognition of the almond flavor in the pastry and the lime blossom flavor in the tea, but also a more immersive, thicker appreciation of the overall experience to which these flavors contribute. To wit, recollections become more vivid and our interpretations of them more meaningful. It is through repeatedly stepping back to recognize and scrutinize categories that we appreciate the kinds of experiences from which the categories emerge. The more attention to categories and their contents the more ripe the practice is for discovering unexpected categories that improve the overall experience: e.g., that heavy silverware can improve flavor (Michel et al., 2015). However, this attention is not necessary to enjoy food. This fact, the extra work required, perceptions that this is finicky and pretentious and worries attending to the parts potentially detracting from the total experience are perhaps why people do not pursue palate development. Likewise, mapping sentences can seem like finicky extra work. In both cases novelty and perceived non-essentiality may cause skepticism. Another potential hurdle is the need for repeated uses of facet theory and mapping sentence correction before theory development and theory extension become truly kinetic. It is precisely this systematic self-examination that results in research that is comparable to and cumulative with other research using the same mapping sentence developed through new mistakes and adjustments to a new context.

Returning to the palate development analogy, repeated exposure to initially unliked flavors may be found to have compelling complexities in the same way do new interpretations of data. Likewise, “humility is an acquired taste” (Roberts, 2014, p. 75) and the reflexivity of a mapping sentence built into the systematic methodology prescribed by facet theory compels the researcher to ask in what ways the collected data gels or does not gel with the mapping sentence's facets; that is, to what extent the hypothesis about the categorial breakdown of the domain in the mapping sentence is respondent-appropriate and relevant to how an individual actually “understands” their experience within the domain's complex context. Assessing both segments of human life and also the totality of the experience allows comparisons between people that speak to enlightening patterns undistorted by a researcher's potential presumptions about the individuals in question.

Hackett does not use words like “humble” nor does he suggest that facet theory might have moral elements in practice. This perhaps was a lost opportunity since a moral dimension would fit into Hackett's philosophical treatment as well as bringing facet theory closer to everyday concerns. Including more descriptive details and specifics—when speaking of the mapping sentence's success in therapy, for instance—would have served a similar purpose. In the same vein, some might find Hackett's examples too niche to give the attention necessary to be convinced of facet theory's merits in that context. This would be a loss, however, because Hackett's examples of what a mapping sentence can do are born out as organic extensions of what facet theory reveals to us about reality and what mapping sentences are for.

Since facet theory is most powerful over long periods of time involving many people in different contexts one test of Hackett's insights and meaning-making will be their echoes in the future in the form of other insights, meaning-making, and perhaps more importantly, as ways to think about domains that map out a way to investigate them. What is needed are more people who believe in facet theory's potential to be applied in a variety of domains. In the short-term, Hackett has achieved one of the most important feats the proponent of a theory can hope for: stimulating well-founded enthusiasm for its adoption and possibilities.

## Author Contributions

The author confirms being the sole contributor of this work and has approved it for publication.

### Conflict of Interest Statement

The author declares that the research was conducted in the absence of any commercial or financial relationships that could be construed as a potential conflict of interest.
